# Investigating the feasibility of generating dual‐energy CT from one 120‐kVp CT scan: a phantom study

**DOI:** 10.1002/acm2.13148

**Published:** 2020-10-14

**Authors:** Wen‐Hui Huang, Kai‐Jie Jhan, Ching‐Ching Yang

**Affiliations:** ^1^ Department of Radiology Mackay Memorial Hospital Taipei Taiwan; ^2^ Department of Biomedical Imaging and Radiological Sciences National Yang‐Ming University Taipei Taiwan; ^3^ Department of Nuclear Medicine National Taiwan University Cancer Center Taipei Taiwan; ^4^ Department of Medical Imaging and Radiological Sciences Kaohsiung Medical University Kaohsiung Taiwan; ^5^ Department of Medical Research Kaohsiung Medical University Chung‐Ho Memorial Hospital Kaohsiung Taiwan

**Keywords:** deep learning, dual energy CT, pseudo CT

## Abstract

**Introduction:**

This study aimed to investigate the feasibility of generating pseudo dual‐energy CT (DECT) from one 120‐kVp CT by using convolutional neural network (CNN) to derive additional information for quantitative image analysis through phantom study.

**Methods:**

Dual‐energy scans (80/140 kVp) and single‐energy scans (120 kVp) were performed for five calibration phantoms and two evaluation phantoms on a dual‐source DECT scanner. The calibration phantoms were used to generate training dataset for CNN optimization, while the evaluation phantoms were used to generate testing dataset. A CNN model which takes 120‐kVp images as input and creates 80/140‐kVp images as output was built, trained, and tested by using Caffe CNN platform. An in‐house software to quantify contrast enhancement and synthesize virtual monochromatic CT (VMCT) for CNN‐generated pseudo DECT was implemented and evaluated.

**Results:**

The CT numbers in 80‐kVp pseudo images generated by CNN are differed from the truth by 11.57, 16.67, 13.92, 12.23, 10.69 HU for syringes filled with iodine concentration of 2.19, 4.38, 8.75, 17.5, 35 mg/ml, respectively. The corresponding results for 140‐kVp CT are 3.09, 9.10, 7.08, 9.81, 7.59 HU. The estimates of iodine concentration calculated based on the proposed method are differed from the truth by 0.104, 0.603, 0.478, 0.698, 0.795 mg/ml for syringes filled with iodine concentration of 2.19, 4.38, 8.75, 17.5, 35 mg/ml, respectively. With regards to image quality enhancement, VMCT synthesized by using pseudo DECT shows the best contrast‐to‐noise ratio at 40 keV.

**Conclusion:**

In conclusion, the proposed method should be a practicable strategy for iodine quantification in contrast enhanced 120‐kVp CT without using specific scanner or scanning procedure.

## 1 Introduction

Single‐energy CT (SECT) scan utilizes a single polychromatic x‐ray beam at energy ranging from 70 to 140 kVp with a standard of 120 kVp. The image contrast of CT depends on the differences in photon attenuation of various materials that constitute human body, whereas the degree of photon attenuation is related to tissue composition and photon energy. Dual‐energy CT (DECT) acquires two images at different energy levels to use the attenuation difference at different energies for deriving additional information, such as virtual monochromatic CT (VMCT) and iodine image.^1^, ^2^, ^3^ The VMCT can be customized to a specific energy level that offers a balance between adequate image contrast and reduced image noise to optimize the contrast‐to‐noise ratio (CNR). Besides image quality enhancement, DECT also allows quantification of iodine concentration, which could improve lesion conspicuity due to difference in iodine content between lesions and normal parenchyma. The algorithms for DECT acquisition are unique for each CT manufacturer, so this capability is only available for some specific scanners.^4^, ^5^ Dual‐source DECT scanners contain two x‐ray tubes and detector arrays for simultaneous acquisition of projection data with the sources operated at different tube potentials. Fast kilovolt‐switching DECT scanners allow acquisition of dual‐energy data by modulating the voltage of a single x‐ray generator from low to high kilovolt peaks between alternating projections. Dual‐layered DECT scanners have equipped with a modified detector with two scintillation layers to receive separate high and low image data. All these proprietary techniques have posed a burden on CT system hardware, so DECT scanners are not widely available as SECT scanner. Moreover, DECT acquisition may increase the radiation dose to patients. Hence, DECT is not a routine procedure even for contrast‐enhanced CT scan in our hospital. Machine learning is attracting growing interest in both academia and industry recently. Furthermore, deep learning techniques have become the de facto standard for a wide variety of computer vision problems.^6^, ^7^, ^8^ A deep learning model learns multiple levels of representations that correspond to different levels of abstraction from the input image to perform prediction. This study aimed to investigate the feasibility of generating pseudo DECT from one 120‐kVp CT by using convolutional neural network (CNN) to derive additional information for quantitative image analysis without extra CT scans through phantom study.

## 2 Methods

### 2.A Calibration phantoms

**Fig. 1:**
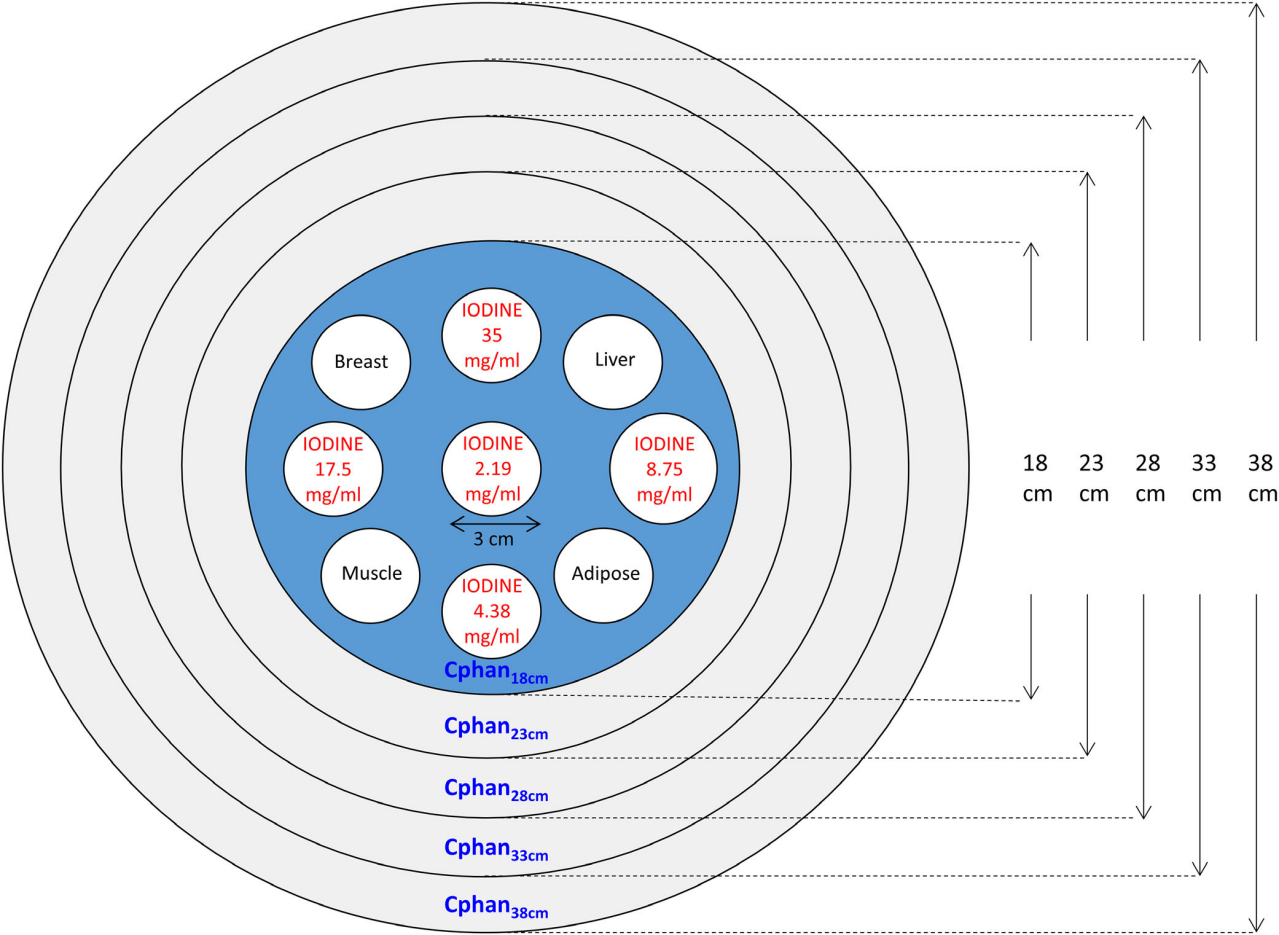
Illustration of the calibration phantoms with five different sizes.

A calibration phantom set which consists of an electron density phantom and additional annuluses was used to generate training dataset for CNN optimization (Fig. 1). The electron density phantom (Model 062; CIRS, Norfolk, VA, USA) which is 18 cm in diameter and 5 cm in height was covered by four layers of 2.5‐cm‐thick bolus (Superflab Bolus; Radiation Products Design Inc, Albertville, MN, USA) to enlarge the diameter of the calibration phantom from 18 cm (Cphan_18cm_) to 23 cm (Cphan_23cm_), 28 cm (Cphan_28cm_), 33 cm (Cphan_33cm_), and 38 cm (Cphan_38cm_). The electron density phantom is made of soft tissue equivalent epoxy resin and houses 4 rod inserts + 5 syringes. The rod inserts simulate four different soft tissues, including adipose (0.96 g/cc), breast (0.991 g/cc), muscle (1.062 g/cc), and liver (1.072 g/cc). The plastic syringes (volume: 10 ml; diameter: 2 cm) were filled with iodine solution at concentration of 2.19, 4.38, 8.75, 17.5, 35 mg/ml.

### 2.B Evaluation phantoms

**Fig. 2:**
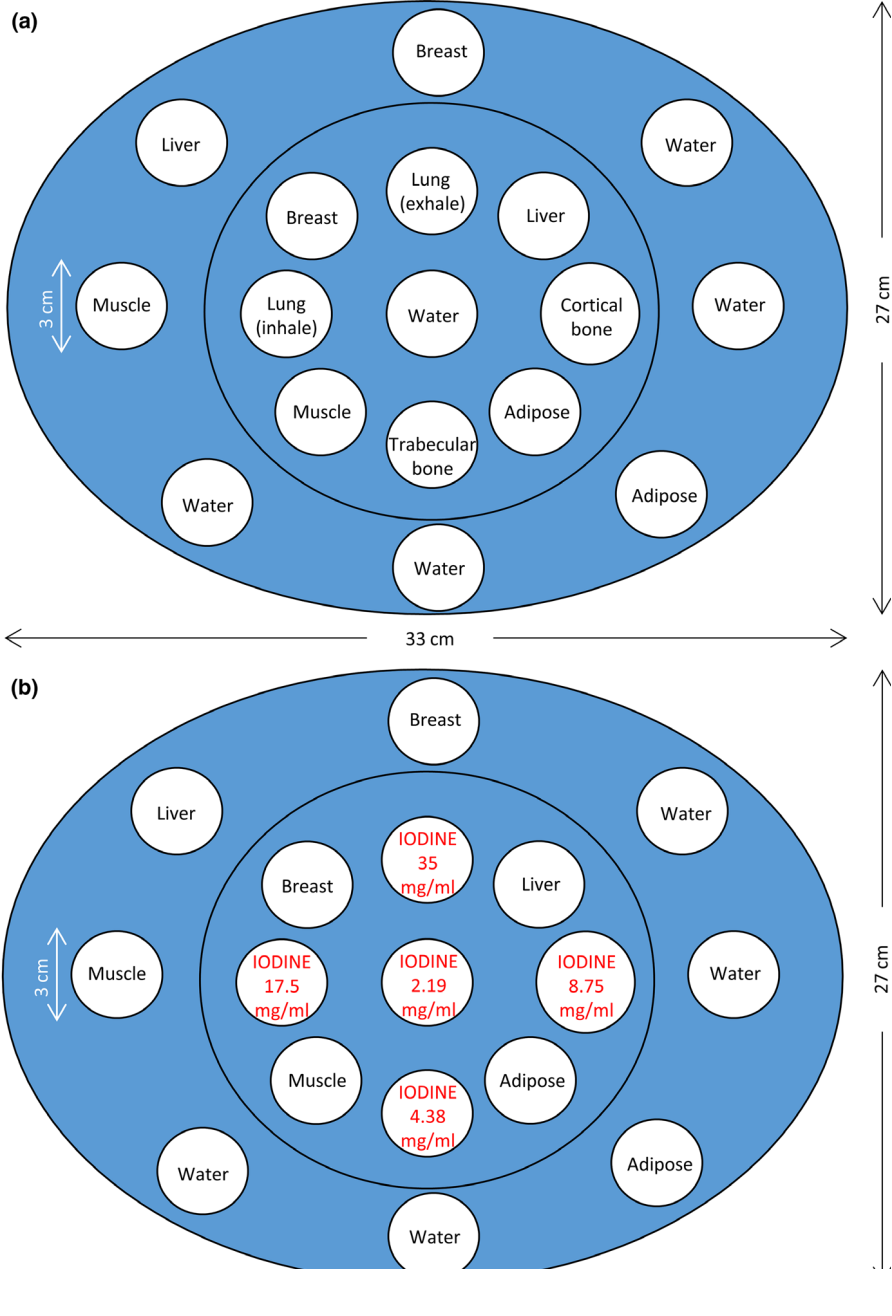
Illustration of two evaluation phantoms with different rod inserts.

Fig. 2 demonstrates two evaluation phantoms used to generate testing dataset for CNN optimization. The first evaluation phantom (Ephan1) shown in [Fig. 2(a)] is an electron density phantom (Model 062; CIRS, Norfolk, VA, USA) with dimensions of 33*27*15 cm^3^. The elliptical, epoxy resin‐based phantom houses 17 rod inserts simulating lung (inhale: 0.195 g/cc; exhale: 0.51 g/cc), adipose (0.96 g/cc), breast (0.991 g/cc), plastic water (1.016 g/cc), muscle (1.062 g/cc), liver (1.072 g/cc), trabecular bone (1.161 g/cc), dense bone (1.53 g/cc). The second evaluation phantom (Ephan2) shown in [Fig. 2(b)] has the same dimensions and base material as Ephan1, but the inserts in Ephan2 are different from those in Ephan1, including 12 rod inserts simulating different tissues and five syringes filled with iodine solution.

### 2.C DECT and SECT scans

All scans were performed on a dual‐source DECT scanner (Somatom Definition Flash, Siemens Healthcare, Forchheim, Germany). The imaging parameters of DECT and SECT scans used in this study are shown in Table 1. Attenuation‐based tube current modulation (CARE Dose 4D, Siemens Healthcare, Forchheim, Germany) was applied for all acquisitions. Each phantom was scanned at 80, 120, and 140 kVp using 0.5‐s gantry rotation time and pitch of 0.6, so 21 CT acquisitions were performed. Scan data were reconstructed at 2‐mm nominal slice width using Filtered Backprojection (FBP) with a medium smooth reconstruction kernel (B30f). For DECT scan, material‐specific images can be generated through material decomposition to quantify the presence of particular elements, compounds, or mixture. With vender’s software, material decomposition was performed for three basis materials in the image domain.^9^, ^10^

**Table 1:** Imaging parameters of dual‐energy and single‐energy scans.

	Dual‐energy Scan		Single‐energy Scan
Tube voltage (kVp)	80	Sn 140	120
Effective tube current‐time product (mAs)^a^	282	120	175
CTDI_vol_ (mGy)^b^	12.13		11.83
Field of view (cm)	50	33	50

^a^ Effective tube current‐time product = mAs/pitch.

### 2.D Deep learning to generate pseudo DECT

**Fig. 3:**
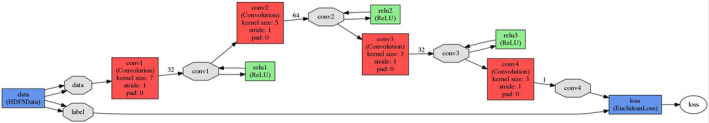
Structure of CNN model to generate pseudo 80‐ and 140‐kVp CT from 120‐kVp CT.

Energy mapping has been widely used in CT‐based attenuation correction for PET which derives μ‐map at 511 keV from CT images. Although this transformation is not linear, it needs small extent of nonlinear mapping.^11^ Hence, the deep learning method proposed by Nie et al. was adapted in this work to generate pseudo DECT imaging from one 120‐kVp CT scan.^12^ Figure 3 demonstrates the structure of the CNN model. The model consists of three convolutional stages with deeply supervised nets (DSN) to supervise features at each convolutional stage, enabled by layer‐wise dense connections in both backbone networks and prediction layers.^13^ The mean square error (MSE) was used as the loss function to minimize the loss between the reconstructed images and the corresponding ground truth. Using MSE as the loss function favors a high peak signal‐to‐noise ratio (PSNR). The input images are prepared as 32*32‐pixel sub‐images randomly cropped from the original image. To avoid border effects, all the convolutional layers have no padding, and the network produces an output image with 18*18 matrix size. The training datasets are sub‐images extracted from the CT images of Cphan_18cm_, Cphan_23cm_, Cphan_28cm_, Cphan_33cm_, Cphan_38cm_ with a stride of 14. The testing datasets are sub‐images extracted from the CT images of Ephan1 and Ephan2 with a stride of 20. The training and testing datasets provide roughly 49972 and 8184 sub‐images, respectively. The filter weights of each layer are initialized by using Xavier initialization, which could automatically determine the scale of initialization based on the number of input and output neurons.^14^ All biases were initialized with zero. The model was trained using stochastic gradient descent with mini‐batch size of 128, learning rate of 0.01 and momentum of 0.9. The CNN model was built, trained, and tested by using Caffe (Convolutional Architecture for Fast Feature Embedding) CNN platform (version 1.0.0‐rc5 with CUDA 8.0.61) on an Ubuntu server (version 16.04.4 LTS) with two RTX 2080 (NVIDIA) graphics cards.

### 2.E In‐house software to generate VMCT and iodine image

**Fig. 4:**
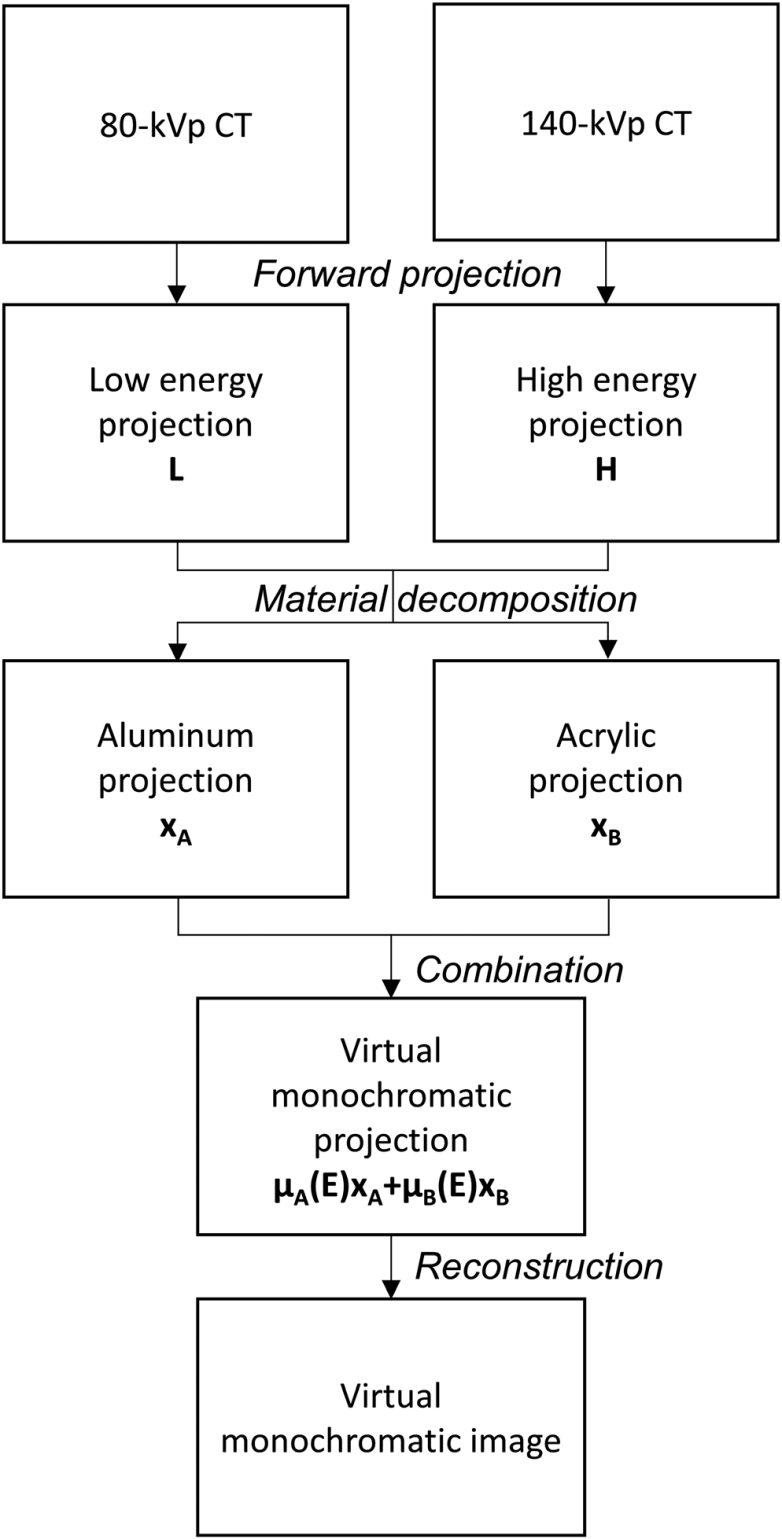
Workflow to synthesize VMCT based on the in‐house software.

In the presence of iodine, VMCT created using image‐based method may contain beam‐hardening artifacts,^15^, ^16^ so an in‐house software for realizing the projection‐based method proposed by Li et al. was implemented (Fig. 4).^17^ The first step in the workflow was forward projection of CT images reconstructed in mm^‐1^ by Siddon’s ray tracing algorithm to obtain low‐energy projections (L) and high‐energy projections (H).^18^ Next, two‐material decomposition was performed to estimate the equivalent thickness of basis materials. Numerous basis materials for soft and bone tissues have been suggested.^19^, ^20^ For this study, aluminum was selected for bone tissues, while acrylic was chosen for soft tissue. The equivalent thicknesses of aluminum (x_A_) and acrylic (x_B_) were estimated based on the following equations:

(1) $$x_{A}=\frac{a_{0}+a_{1}L+a_{2}H+a_{3}L^{2}+a_{4}\text{LH}+a_{5}H^{2}}{1+b_{0}L+b_{1}H}$$

(2) $$x_{B}=\frac{c_{0}+c_{1}L+c_{2}H+c_{3}L^{2}+c_{4}\text{LH}+c_{5}H^{2}}{1+d_{0}L+d_{1}H}$$

where the parameters a_i_, b_j_ c_i_, d_j_ (i = 0‐5; j = 0, 1) represent characteristics of the x‐ray beam energy spectrum. In the combination step, virtual monochromatic projections were synthesized using the following equation:

(3) $$\int \mu \left(E\right)\text{ds}=\mu_{A}\left(E\right)x_{A}+\mu_{B}\left(E\right)x_{B}$$

where μ_A_(E) and μ_B_(E) are the linear attenuation coefficients of basis materials at energy E. The mass attenuation coefficients of basis materials at different energies were obtained from XCOM: Photon Cross Sections Database by National Institute of Standards and Technology (NIST), available at http://physics.nist.gov/xcom. Last, FBP algorithm was used for the VMCT reconstruction. With regards to the estimation of iodine concentration, the decomposed aluminum projections were reconstructed by FBP first and then multiplied by a conversion factor to create iodine images.

The parameters a_i_, b_j_ c_i_, d_j_ in [Eq. (1) and (2)] have to be determined to conduct material decomposition. Hence, a calibration step wedge which contains two aluminum step wedges and one acrylic step wedge stacked in an orthogonal pattern was used. The dimensions of the 11‐step aluminum wedge (Fluke Biomedical, Everett, WA, USA) are 139.7 mm in length, 63.5 mm in width and 33 mm in height. The home‐made acrylic wedge contains eight steps, and its dimensions are 120 mm in length, 152.4 mm in width and 40 mm in height. Forty‐eight regions of interest (ROIs) were placed on the projections of the calibration step wedge (x_A_: 0, 6, 12, 18, 24, 30 mm; x_B_: 10, 15, 20, 25, 30, 35, 40 mm) to determine image intensity in L and H. Given x_A_, x_B_ and their corresponding image intensity in L and H, the parameters a_i_, b_j_ c_i_, d_j_ can be determined by minimizing absolute error fitting. This step is called parameterization. To validate the results of parameterization, the thicknesses of aluminum and acrylic step wedges estimated based on [Eq. (1) and (2)] were compared with those measured using a caliber.

### 2.F Quantitative evaluation

The difference between real CT images (I_real_) and pseudo CT images (I_pseudo_) generated by CNN was quantified by using RMSE and PSNR:

(4) $$\text{RMSE}=\sqrt{\frac{\sum_{i=1}^{V}{\left(I_{\text{real}}‐I_{\text{pseudo}}\right)}^{2}}{V}}$$

where V is the number of voxels within the whole image,

(5) $$\text{PSNR}=20{\text{log}}_{10}\frac{I_{\text{max}}}{\text{RMSE}}$$

(6) $$\text{CNR}=\left|\frac{\text{CT}\#‐\text{CT}{\#}_{\text{BG}}}{{\text{SD}}_{\text{BG}}}\right|$$

where CT# is the mean CT number of a specified material, CT#_BG_ and SD_BG_ are the average and standard deviation of CT numbers of tissue equivalent background material, respectively.

## 3 Results

### 3.A Parameterization for material decomposition

According to the calibration step wedge experiment, the parameters a_i_, b_j_ c_i_, d_j_ in [Eq. (1) and (2)] were determined:

$$x_{A}=\frac{0.952+1.116L‐2.353H‐0.023L^{2}+0.098\text{LH}‐0.104H^{2}}{1‐0.020L+0.042H}$$

$${\;x}_{B}=\frac{‐2.319‐2.882L+8.509H+0.088L^{2}‐0.448\text{LH}+0.558H^{2}}{1‐0.035L+0.079H}.$$

**Fig. 5:**
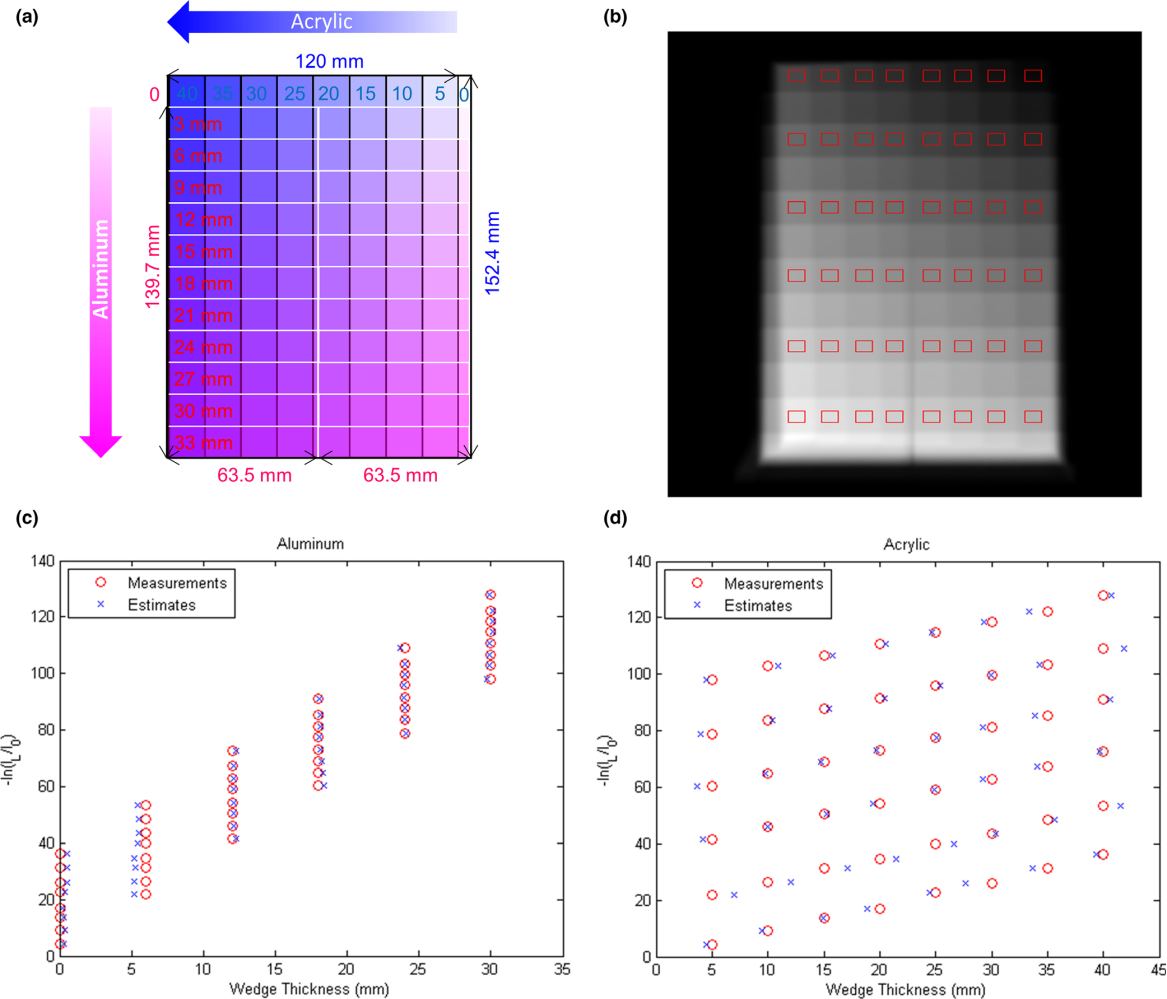
(a) Illustration of the calibration step wedge and (b) the corresponding 80‐kVp projection. The red rectangles in (b) are the ROIs used to depict the image intensity in projection versus the wedge thickness of (c) aluminum step wedge and (d) acrylic step wedge.

**Fig. 6:**
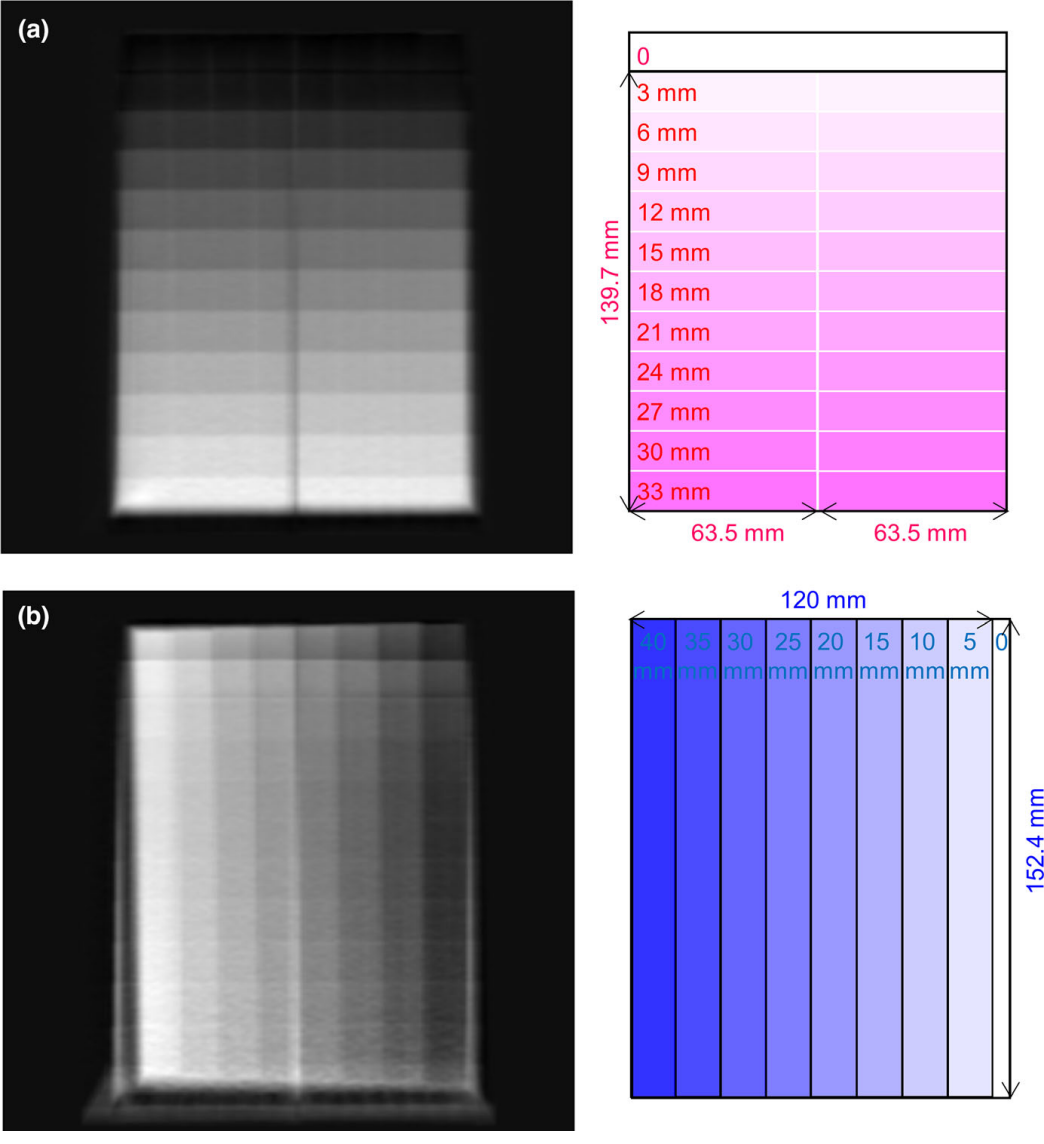
Decomposed projection from basis material decomposition (left) and the corresponding illustration (right) for (a) aluminum step wedge and (b) acrylic step wedge.

Figures 5(a) and 5(b) demonstrate the illustration of the calibration step wedge and the corresponding 80‐kVp projection with 48 ROIs, respectively. The measured and estimated wedge thickness vs the image intensity in 80‐kVp projection (‐ln(I_L_/I_0_)) are shown in [Fig. 5(c)] for aluminum step wedge and [Fig. 5(d)] for acrylic step wedge. The differences between measurements and estimates for the aluminum step wedge with thickness of 0, 6, 12, 18, 24, 30 mm are 0.39, 0.66, 0.15, 0.19, 0.07, 0.14 mm, respectively. The differences between measurements and estimates for the acrylic step wedge with thickness of 5, 10, 15, 20, 25, 30, 35, 40 mm are 0.99, 0.88, 1.48, 0.42, 0.54, 0.76, 0.77, 0.61 mm, respectively. Figure 6 demonstrates the decomposed projections from basis material decomposition for aluminum and acrylic step wedges and their corresponding illustrations.

### 3.B Real DECT images + in‐house software

**Fig. 7:**
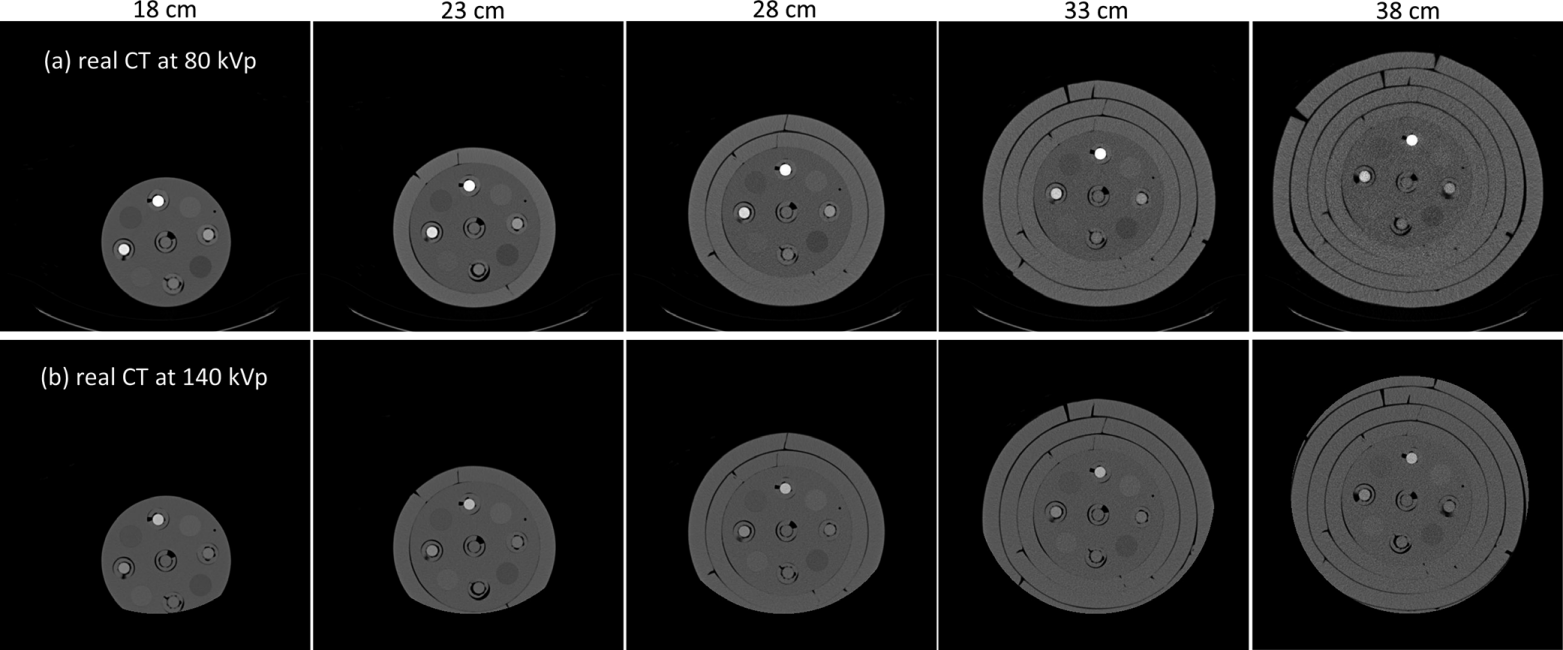
(a) 80‐kVp and (b) 140‐kVp real CT images of the calibration phantoms with five different sizes (window width/window level = 1600/0 HU).

**Fig. 8:**
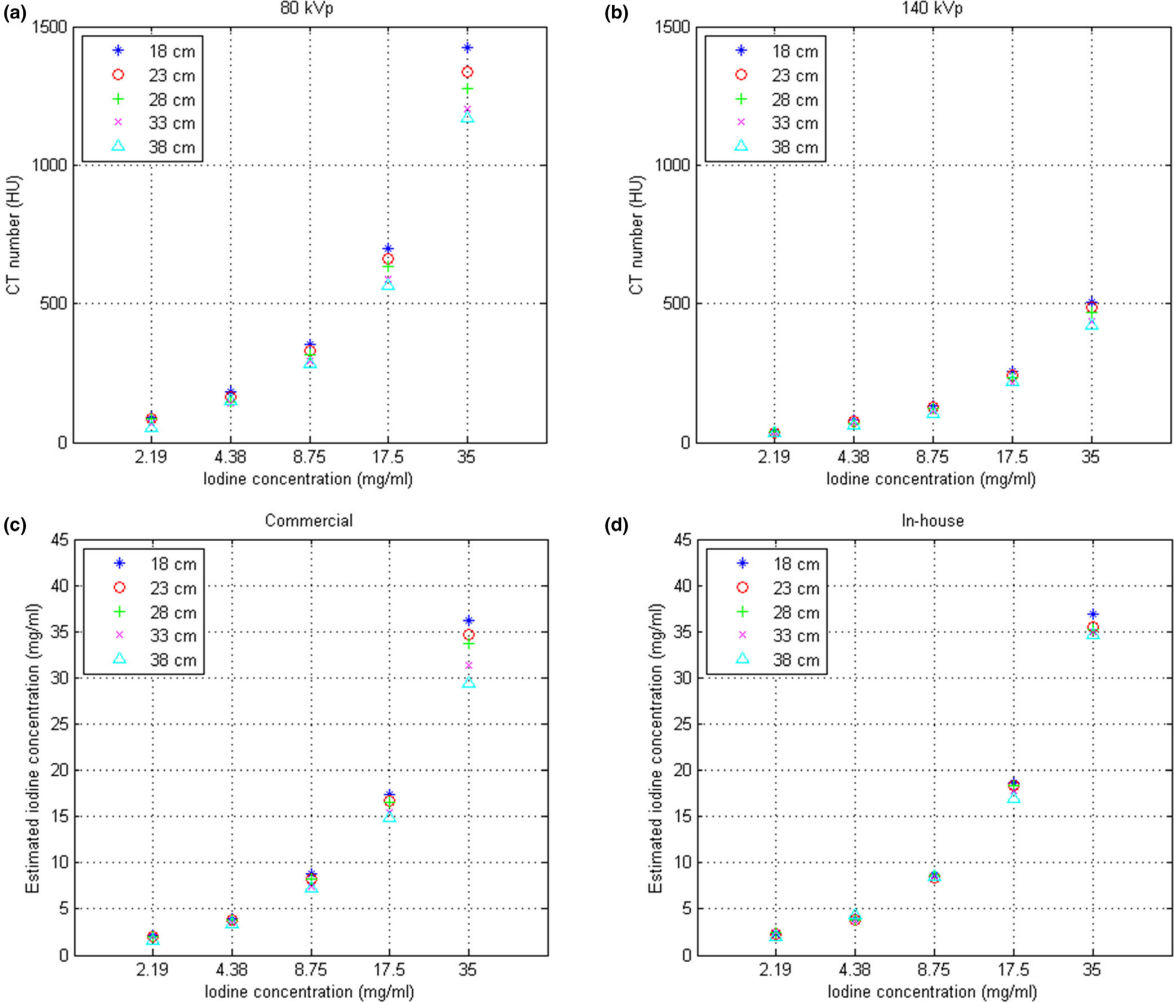
CT numbers of (a) real 80‐kVp and (b) real 140‐kVp images and iodine concentration estimated based on (c) commercial software and (d) in‐house software with real DECT for iodine syringes inserted in the calibration phantoms with five different sizes.

Figure 7 shows CT images of Cphan_18cm_, Cphan_23cm_, Cphan_28cm_, Cphan_33cm_, Cphan_38cm_ obtained from real DECT scans. For these acquired images, the CT numbers of iodine syringes at 80 and 140 kVp are depicted in [Figs. 8(a) and 8(b)], respectively, and their iodine concentrations estimated by commercial and in‐house software are depicted in [Figs. 8(c) and 8(d)], respectively. The coefficients of variation (CVs) of CT numbers at 80 kVp due to different phantom sizes are 0.196, 0.099, 0.085, 0.081 and 0.076 for syringes filled with iodine concentration of 2.19, 4.38, 8.75, 17.5, 35 mg/ml, respectively. The corresponding CVs for CT numbers at 140 kVp are 0.252, 0.100, 0.075, 0.075, 0.072. With regards to the iodine concentration estimated by the commercial software, the estimates averaged over different phantom sizes are differed from the truth by 0.202, 0.662, 0.784, 1.310, 2.430 mg/ml for syringes filled with iodine concentration of 2.19, 4.38, 8.75, 17.5, 35 mg/ml, respectively. The corresponding results for iodine concentration estimated by the in‐house software are 0.276, 0.316, 0.188, 0.574, 0.588 mg/ml.

### 3.C Pseudo DECT images + in‐house software

**Fig. 9:**
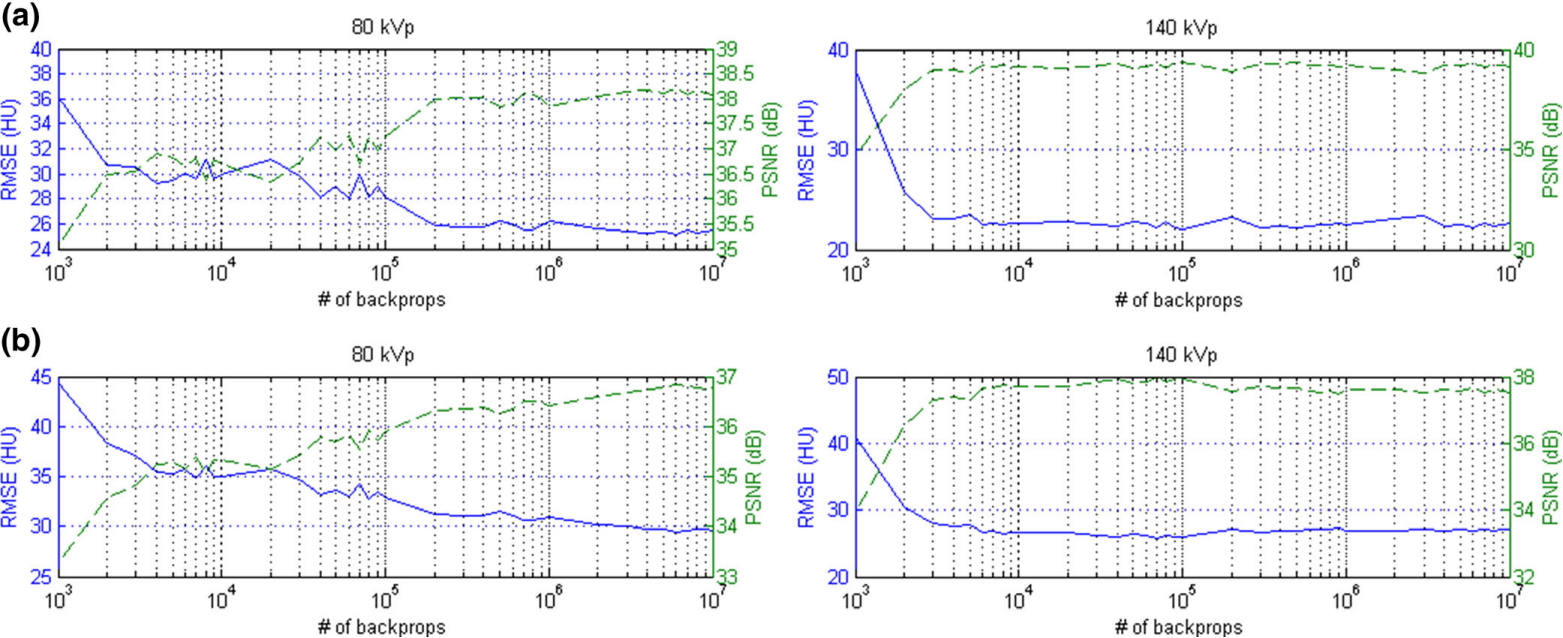
RMSE (left vertical axis, solid blue line) and PSNR (right vertical axis, dashed green line) between real and pseudo CT at 80 kVp (left) and 140 kVp (right) for (a) Ephan1 and (b) Ephan2.

**Fig. 10:**
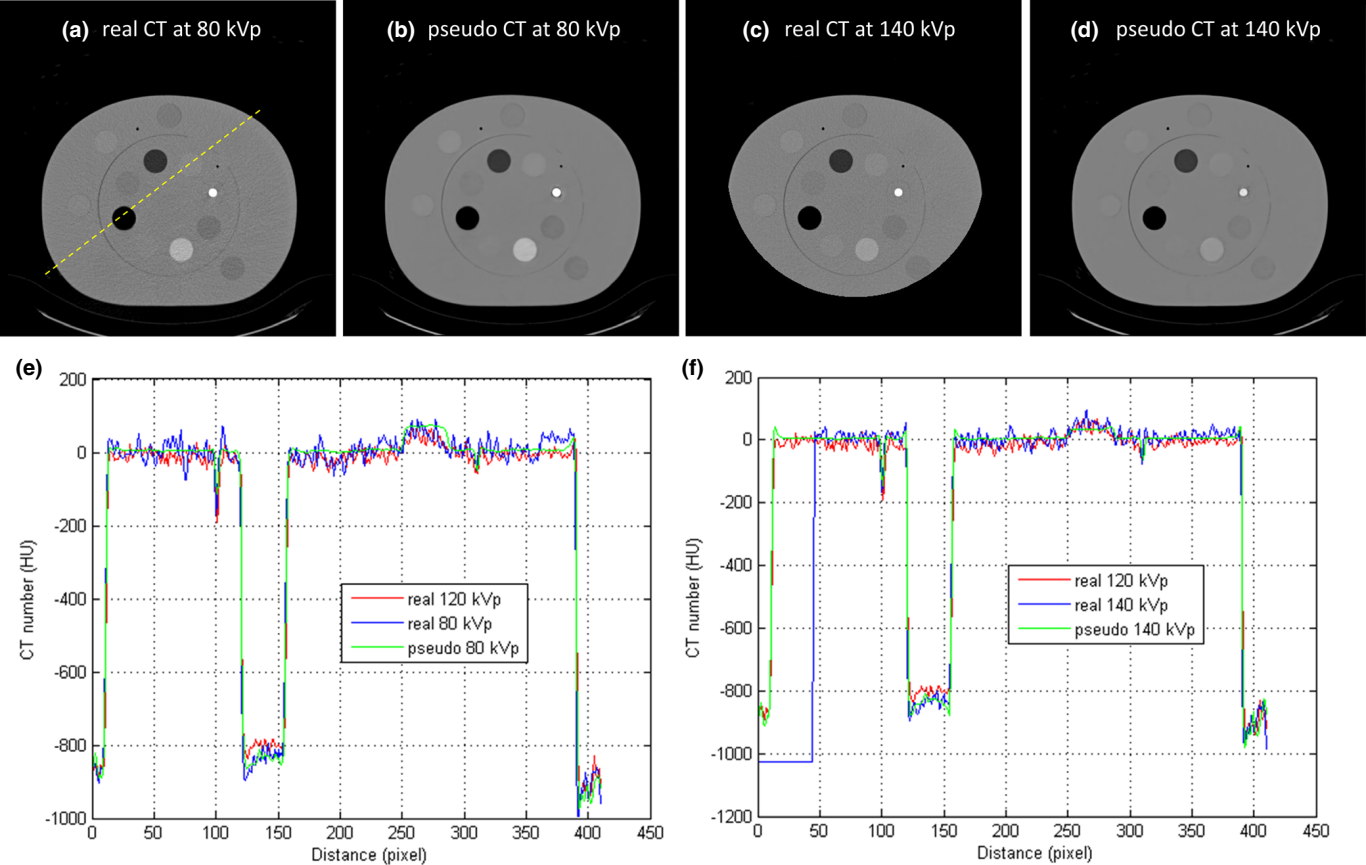
(a) Real 80‐kVp CT image, (b) pseudo 80‐kVp CT image, (c) real 140‐kVp CT image, (d) pseudo 140‐kVp CT image for Ephan1 (window width/window level = 1600/0 HU). Intensity profiles of SECT, real and pseudo DECT through the dashed line in (a) are compared in (e) for 80‐kVp images and (f) for 140‐kVp images.

**Fig. 11:**
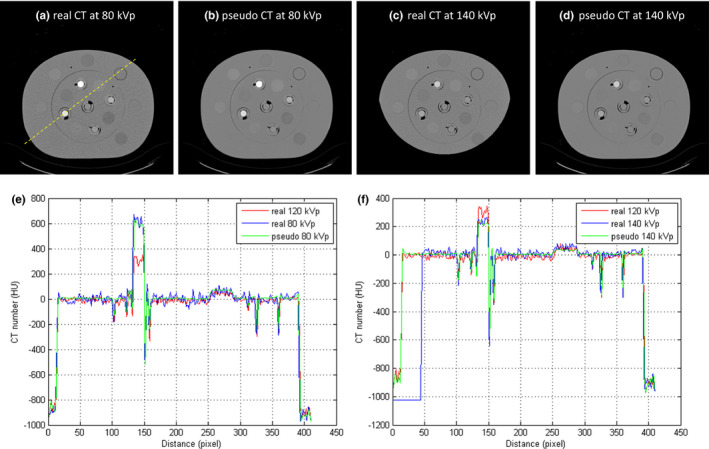
(a) Real 80‐kVp CT image, (b) pseudo 80‐kVp CT image, (c) real 140‐kVp CT image, (d) pseudo 140‐kVp CT image for Ephan2 (window width/window level = 1600/0 HU). Intensity profiles of SECT, real and pseudo DECT through the dashed line in (a) are compared in (e) for 80‐kVp images and (f) for 140‐kVp images.

**Fig. 12:**
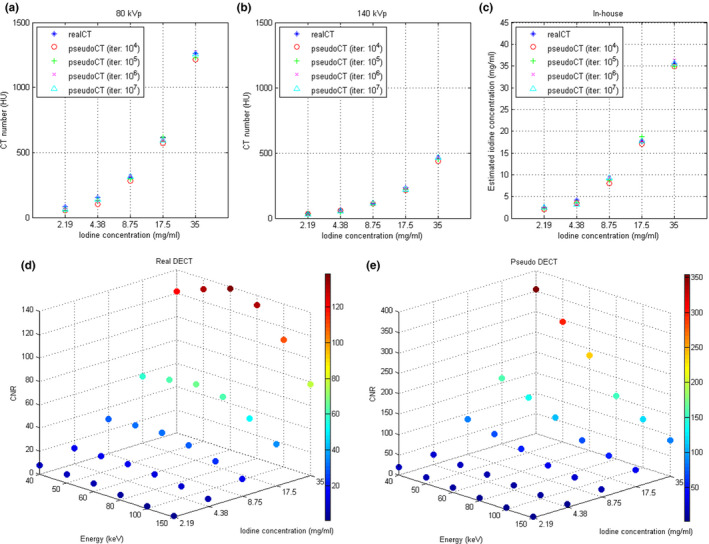
CT numbers at (a) 80 kVp and (b) 140 kVp, (c) iodine concentration estimated based on in‐house software, and CNR of VMCT synthesized by in‐house software with (d) real DECT and (e) pseudo DECT for iodine syringes inserted in Ephan2.

Figure 9 demonstrates the RMSE and PSNR between real and pseudo CT for Ephan1 and Ephan2. The pseudo DECT images of Ephan1 generated by CNN after 10^7^ iterations are compared with real DECT images in Fig. 10. The corresponding results for Ephan2 are shown in Fig. 11. Since the field of view (FOV) in 140‐kVp scan is 33 cm, the peripheral parts of evaluation phantoms are truncated in real 140‐kVp CT image [Figs. 10(c) and 11(c)]. For a fair comparison between 80‐ and 140‐kVp images in terms of RMSE and PSNR, the difference images were masked by a binary image which is the union of phantom boundary and the 33‐cm FOV. To evaluate the efficacy of our proposed method on quantification accuracy, the CT numbers of iodine syringes at 80 and 140 kVp are depicted in [Figs. 12(a) and 12(b)], and the iodine concentrations estimated by the in‐house software are depicted in [Figs. 12(c)]. For 80‐kVp CT, the CT numbers in pseudo images generated by CNN after 10^7^ iterations are differed from the truth by 11.57, 16.67, 13.92, 12.23, 10.69 HU for syringes filled with iodine concentration of 2.19, 4.38, 8.75, 17.5, 35 mg/ml, respectively. The corresponding results for 140‐kVp CT are 3.09, 9.10, 7.08, 9.81, 7.59 HU. As for the iodine concentration estimated by the in‐house software with pseudo DECT generated by CNN after 10^7^ iterations, the estimates are differed from the truth by 0.104, 0.603, 0.478, 0.698, 0.795 mg/ml for syringes filled with iodine concentration of 2.19, 4.38, 8.75, 17.5, 35 mg/ml, respectively. With regards to the efficacy of our proposed method on image quality enhancement, the CNR of VMCT synthesized by the in‐house software with real and pseudo DECT are demonstrated in [Figs. 12(d) and 12(e)] for iodine syringes inserted in Ephan2.

## 4 Discussion

Contrast material enhancement for CT has been used since the mid‐1970s. Besides providing visual enhancement between a lesion and the normal surrounding structures, contrast enhanced CT can also be used for estimating iodine concentration through DECT. Since CT contrast enhancement in the lesion has close relationship with the vascular density, the iodine volume is associated with tumor differentiation.^21^ It has been reported that peak enhancement intensity is negatively correlated with tumor differentiation based on the density of immature microvessels.^22^ Determining a scan timing to grab the right moment of maximal contrast differences between a lesion and the normal parenchyma is crucial in contrast enhanced CT. However, the optimal timing varies among patients because it is related to numerous interacting factors, such as cardiac output, venous access, renal function, hepatic cirrhosis, and so on.^23^, ^24^, ^25^ Consequently, the reliability of DECT‐derived iodine concentration for pathologic stage classification may be affected by some of the patient‐related factors. Hence, DECT scan which quantifies iodine concentration at one time point is not used in daily practice for cancer screening and staging in our hospital. For the detection of hepatocellular carcinoma (HCC), dynamic scan which acquires 120‐kVp SECT images to see the enhancement in different phases is used instead. The combination of arterial phase hyperenhancement followed by portal venous phase washout appearance strongly suggests the diagnosis of HCC.^26^ However, triple‐phase CT is a qualitative evaluation method and relies heavily on radiologist’s subjective visual assessment. CT perfusion imaging represents an important quantitative assessment method for tumor‐related vascularization, which can measure the hemodynamic parameters at the capillary level, with high temporal and spatial resolution, as well as good reproducibility.^27^ But the respiratory motion and high radiation dose are major limitations that need to be overcome in order for perfusion CT to be used in clinical settings.

In this work, the feasibility of using deep learning method to generate pseudo DECT based on one 120‐kVp SECT scan for quantitative image analysis has been investigated through phantom study. According to Fig. 8, CT numbers for the same iodine syringe vary with phantom size, which was also observed in estimated iodine concentrations. Nevertheless, the estimation accuracy of in‐house software was comparable to that of commercial software for real DECT imaging. Due to beam hardening, a lower CT number was observed in a larger calibration phantom for the same iodine syringe.^28^ This phenomenon could increase data diversity to improve CNN’s generalization accuracy. As shown in Fig. 9, the RMSE between real and pseudo CT was slightly lower in Ephan1 than that in Ephan2, although the rod inserts in Ephan1 simulating inhale lung, exhale lung, trabecular bone and dense bone are not included in the calibration phantoms. The intensity profiles shown in Figs. 10 and 11 and the CT numbers shown in [Figs. 12(a) and 12(b)] also verify the effectiveness of the investigated CNN model in energy mapping. Consequently, the estimation accuracy of in‐house software with CNN‐generated pseudo DECT was comparable to that with real DECT. Besides estimating iodine volume, the proposed method also creates VMCT. VMCT allows for reconstruction of images at different energies, so it could offer better image contrast than 120‐kVp SECT scans after energy optimization. Lowering energy could improve image contrast but would also increase image noise.^29^ For VMCT synthesized by using real DECT, the best CNR was found in 60‐keV images. However, VMCT synthesized by using pseudo DECT shows the best CNR at 40 keV. Based on our results, the difference in CT number between real and pseudo CT was little, but the image noise in pseudo CT is much lower than that in real CT (see intensity profiles in Fig. 10 and 11). The difference in noise properties between real and pseudo CT propagates to the corresponding VMCT, which may explain the difference in CNR performance shown in [Figs. 12(d) and 12(e)]. Overall, the proposed method should be a practicable workflow for iodine quantification in contrast enhanced 120‐kVp SECT without using specific scanner or scanning procedure.

Several limitations to this study need to be acknowledged. First, the data acquisition, processing and reconstruction approaches can influence the study results. The protocol parameters used in this study are suggested by the manufacturers and are currently employed in many centers equipped with the same scanners. Additional studies assessing the proposed workflow for different DECT scanners will be needed and valuable. Second, CT images were acquired either with the calibration phantoms or with the evaluation phantoms. When the proposed workflow is translated to clinical use, it is expected that the accuracy of CNN‐generated pseudo DECT determines the performance of the proposed method. Challenges arise because tissue heterogeneity is not modeled in this phantom study. In clinical implementation, transfer learning should be performed to retrain the CNN model by using CT images obtained from patient DECT and SECT scans. The efficacy of the proposed workflow on clinical patient data needs to be further investigated.

## 5 Conclusion

This study investigated the feasibility of generating pseudo DECT from one 120‐kVp CT by using deep learning method to quantify iodine concentration and synthesize VMCT through phantom study. Based on our results, the accuracy of iodine concentration estimated by the in‐house software with CNN‐generated pseudo DECT imaging was comparable to the commercial software with real DECT imaging. Moreover, the VMCT synthesized by the proposed method could provide better image contrast than 120‐kVp SECT after energy optimization. In conclusion, the proposed method should be a practicable strategy for iodine quantification in contrast enhanced 120‐kVp SECT without using specific scanner or scanning procedure.
